# Availability of family care resources, bathing assistance and toileting assistance among older adults with functional limitations: an evidence-based study from China

**DOI:** 10.1186/s12877-024-05047-5

**Published:** 2024-05-11

**Authors:** Jinxin Zhang, Zi Shen, Xiyang Tong, Xiaojie Sun, Nengliang Yao

**Affiliations:** 1 Centre for Health Management and Policy Research, School of Public Health, Cheeloo College of Medicine, Shandong University, 44 Wenhuaxi Rd, Jinan, Shandong 250012 China; 2 NHC Key Lab of Health Economics and Policy Research, Shandong University, Jinan, 250012 China; 3 Zibo First Hospital, Zibo, Shandong 255200 China; 4 Home Centered Care Institute, Schaumburg, IL USA; 5 University of Virginia, Charlottesville, VA USA

**Keywords:** Family Care, Bathing, Toileting, China

## Abstract

**Background:**

An aging population has contributed to an increasing prevalence of functional limitations among older adults. Family support plays a crucial role in toileting and bathing assistance. Yet, the relationship between availability of family care resources and such actual assistance remains insufficiently explored. Our study aims to describe availability of family care resources and identify the association between availability of family care resources and toileting assistance or bathing assistance.

**Methods:**

This study employed a cross-sectional analysis of data from the 2018 National Survey of the China Health and Retirement Longitudinal Study (CHARLS). The availability of family care resources was assessed using measurements of spouse availability, adult child availability, and living arrangement. Bathing assistance and toileting assistance were measured based on self-reported receipt of such assistance. Descriptive statistics were used to depict the overall and subgroup situation of availability of family care resources. Multivariable logistic models were employed to investigate the relationship between availability of family care resources and the receipt of toileting assistance or bathing assistance.

**Results:**

Among the sample of older adults with functional limitations, 69% had a spouse, 63% had at least one adult child, and 80% resided with family members. Among those with bathing disability, 13% reported lacking bathing assistance, and among those with toileting disability, 54% reported lacking toileting assistance.

Participants with 1-2 adult children had lower odds of receiving toileting assistance (OR: 0.28, 95% CI: 0.09, 0.91, *p*= 0.034) compared to those with three or more adult children. Spouse availability and living arrangement did not exhibit statistically significant associations with toileting assistance. Participants without a spouse had lower odds of receiving bathing assistance (OR: 0.27, 95% CI: 0.09-0.78, *p*= 0.016) in comparison to those with a spouse; however, adult child availability and living arrangement did not display statistically significant associations with bathing assistance.

**Conclusion:**

The present findings suggest a gap in family commitment when it comes to assisting older adults with functional limitations in bathing/toileting. To address this, policymakers are encouraged to prioritize the implementation of proactive mechanisms for identifying family caregivers, alongside incentives to enhance their engagement in practical caregiving activities. Furthermore, it is crucial to emphasize the prioritization of affordable and easily accessible formal toileting/bathing assistance options for older adults who lack sufficient family care resources.

## Introduction

The magnitude of China’s aging phenomenon stands out distinctly in the world. Notably, the total number of older adults with functional limitations in China increased from 33 million to 40 million between 2010 and 2020 [1, 2], and is projected to reach 140 million by 2050 [3]. Activities of daily living and instrumental activities of daily living serve as pivotal indicators of functional limitations for older adults [4]. Activities of daily living focus on self-care activities encompassing bathing, dressing, toileting, transferring, continence, and feeding [5], while instrumental activities of daily living encompass community-related activities like housekeeping, cooking, shopping, managing finances, and medication administration [6].

Among these daily living activities, toileting and bathing deserve attention in later life due to their distinct characteristics. On one hand, bathing serves the purposes of attaining cleanliness, fostering a sense of order and routine in daily existence, and facilitating relaxation and rejuvenation [7]. This practice is generally imbued with values inculcated during youth, often intertwined with concepts of well-being and virtue [7]. On the other hand, the role of toileting is of pronounced significance as a contributing factor to unintentional falls and injuries among older individuals [8]. Paradoxically, for those who require toileting assistance, the act of having their toileting behavior observed by others is often met with discomfort and engenders feelings of embarrassment [9]. Moreover, the privacy inherent to both bathing and toileting activities necessitates a secluded environment. Additionally, both toileting and bathing necessitate fundamental physical coordination, balance, and sensory capabilities. Specifically, toileting activities encompass a spectrum of tasks, including the donning and doffing of clothing, cleansing the buttocks, and dispensing toilet paper [10, 11]. Similarly, bathing involves a repertoire of actions such as bending, turning, lifting limbs, and employing manual dexterity to manipulate objects [12, 13].

Toileting disability or bathing disability is commonly defined as the presence of difficulties in performing toileting or bathing activities, necessitating external support to accomplish these tasks [14, 15]. Previous studies have consistently shown that toileting disability significantly influence mental health, social participation [16], and the quality of life [17]. A prospective cohort study involving community-dwelling older persons revealed an association between persistent bathing disability and the heightened risk of eventual admission to long-term nursing care facilities [18]. Consequently, the provisions of toileting assistance and bathing support hold notable clinical and societal implications of significance.

Concerning care provision, the sustainability of funding for formal care systems serving older adults faces challenges [19]. Guided by Chinese filial piety, which emphasizes family members providing physical care, emotional support, respect, and obedience to older adults, a significant number of older individuals rely on family support. This reliance on family support is expected to persist [3]. Recent Chinese national research on the living arrangements of older adults indicates that 91.7% reside with family members [20]. These cohabitants often serve as primary caregivers for older adults. Moreover, many older individuals have children living with them or nearby, who offer frequent contact and regular non-financial assistance [20, 21]. Additionally, currently married older adults exhibit significantly lower rates of unmet needs compared to those who are single, separated, or divorced [22]. Older adults primarily rely on spouses, adult children, and cohabitants to provide them with care in a home setting, as they serve as crucial family care resources for providing practical assistance [23]. Nevertheless, older individuals with access to family care resources may not always receive care that aligns with their specific needs. Prior studies, such as Zhu (2015), have indicated that the unmet needs for daily care among older individuals have consistently remained high over the years [24]. Additionally, research by Desai (2001), has revealed that the prevalence of unmet needs in specific areas, such as toileting (17.6%) and bathing (16.7%), exceeds that for eating (10.2%) and dressing (13.1%) [25]. This suggests that caregivers may have prioritized assistance with eating and dressing over toileting and bathing [25].

From a perspective of national policy and planning, the current trend in China emphasizes promoting family care as a key component in providing social services to the older people. This approach encompasses a comprehensive framework with three primary levels of care: family-based care, serving as the foundational component; community-based care, functioning as a supportive element; and institutional care, serving as a complementary aspect. Informal care by family members is considered the primary support for Chinese older people within this framework. To facilitate the transformation of available family care resources into practical support and to enhance the development and targeting of home and community services, it is essential to understand the prevalence and the relationship between availability of family care resources and assistance with toileting or bathing.

However, there have been few population-based studies that have investigated the prevalence and the relationship between availability of family care resources and assistance with toileting or bathing. Numerous studies have examined the prevalence of family care providers for older adults with functional limitations [3, 26]; however, they did not adequately examine the prevalence of the availability of family care resources. Some studies have used availability of family care resources (e.g., having adult children) as an instrumental variable to mitigate endogeneity when assessing the effect of informal care on formal care use [27], but not as the primary predictor nor specifically related to toileting assistance or bathing assistance. Moreover, in most studies, receiving family care has been typically examined as a summation of caregiving across various activities such as dressing, bathing, transferring, toileting, and eating.

Providing support to older adults with functional limitations living in the community is a crucial health policy concern in this century. Personal hygiene, particularly bathing and toileting, has historically not received adequate attention [28]. Additionally, it is important to note that activities such as bathing or showering are frequently regarded as highly private. This study presents essential new evidence regarding the association between availability of family care resources and bathing assistance, which can inform policies and interventions aimed at enhancing family care overall. The specific research questions are as follows: What is the status of availability of family care resources (spouse availability, adult child availability, and living arrangement) among adults with functional limitations? Are those with family care resources more likely to receive assistance with bathing and toileting, compared to those without such resources?.

## Methods

### Data and sample

We created two analysis samples based on the data from the China Health and Retirement Longitudinal Study (CHARLS), a comprehensive nationwide survey program with the goal of collecting high-quality data on various aspects related to the social, economic, health behavior, and health outcomes among middle- and old-aged residents in China. The CHARLS survey covered residents living in communities from 450 villages and 150 counties across 28 provinces in China. The sampling process employed a probability-proportional-to-size technique using a sampling frame that included all county-level units except Tibet [29]. The primary objective of CHARLS was to establish a robust and publicly accessible micro-database that contains diverse information, such as demographic characteristics, socioeconomic status, health conditions, and healthcare utilization patterns.

First, we used the CHARLS data surveyed in 2018 to select a disability sample of adults 65 or older who had functional limitations. Functional limitation refers to individuals who report experiencing difficulties in performing at least one Activity of Daily Living (ADL) or Instrumental Activity of Daily Living (IADL) during the interview [5, 6]. The ADL measurement comprised six items, including dressing, eating, bathing or showering, getting in or out of bed, toileting, and controlling urination and defecation [5]. The IADL measurement consisted of five items: housekeeping, cooking, shopping, managing money, and taking medication [6]. For each item, participants were presented with four response options: (1) Activity can be performed without any difficulty; (2) have difficulty but can still do it (3) have difficulty and need help; and (4) unable to complete the activity. Scoring was conducted as follows: zero points were assigned if the participant chose “without any difficulty” or “have difficulty but can still do it,” while one point was given for any other response. Participants who reported not needing help with both ADL and IADL were excluded from the study, as the focus was on individuals with functional limitations. The sample for this study included 2,378 persons. This disability sample was used to provide estimates of availability of family care resources for older people with functional limitations, both overall and for each demographic group.

Second, to examine toileting assistance in relation to availability of family care resources, we limited the sample to individuals who reported having a toileting disability at the time of the interview, resulting in a sub-sample of 226 persons. Similarly, to investigate bathing assistance in relation to availability of family care resources, we limited the sample to individuals who reported having a bathing disability at the time of the interview, resulting in a sub-sample of 377 persons.

### Measures

We selected the availability of family care resources variables that had been previously identified as potentially important factors associated with caregiving [30–33]. For spouse availability, we included the presence of a spouse (couple, single). Regarding the potential availability of adult children, we considered the number of adult children (0, 1–2, ≥ 3 persons). Additionally, we included living arrangements (with others, alone).

An outcome measure was formulated to determine whether individuals with disabilities received toileting assistance or bathing assistance. Receiving bathing assistance was defined as individuals with bathing disability self-reporting that they receive help from someone during bathing. This was assessed through the question, “Does anyone ever help you bathe?” Participants who answered “yes” were labeled as 1, while those who answered “no” were labeled as 0. In a similar manner, toileting assistance was assessed.

The covariates included in the analysis were as follows: gender, age (65-69, 70-74, ≥ 75 years old), education level (lower than primary school, primary school or above), area of residence (rural, urban), engagement in social activities (no, yes), use of home and community-based services (no, yes), walking stick use (no, yes), self-rated health status (healthy, unhealthy), number of chronic diseases (0, 1, ≥ 2), number of items in the difficulty in performing ADL category (1-2, 3-6), and number of items in the difficulty in performing IADL category (0-2, 3-5).

### Analytic approach

The data analysis was performed using Stata 15.0. Descriptive statistics were used to present the percentages for each status of spouse availability, adult child availability, and living arrangements within the overall sample of adults aged 65 and above with functional limitations, as well as within each demographic subgroup (Table 1). Next, chi-square test was used to compare the characteristics for toileting disability with and without toileting assistance (Table 2). The *p* value from the chi-square test for each factor was also provided. Similarly, chi-square test was used to compare the characteristics for disability with and without bathing assistance (Table 3). Additionally, a multivariable logistic model was conducted to assess the association between availability of family care resources and toileting assistance (Table 4). Similarly, a multivariable logistic model was performed to assess the association between availability of family care resources and bathing assistance (Table 5). To evaluate multicollinearity, we calculated the variance inflation factor (VIF), where a VIF value greater than 10 indicates severe multicollinearity. The results were reported in terms of odds ratios (OR) with corresponding 95% confidence intervals (CI) for each variable.

**Table 1:** Availability of family care resources among older adults with functional limitation. (sample: older adults with functional limitations; 2378 persons)

**Factors**	**Total**		**Spousal availability**		**Adult child availability**			**Live arrangement**	
			Couple	Single	0 person	1-2 person(s)	≥3 persons	With others	Alone
**Total**			1,651(69.43)	727(30.57)	884(37.17)	384(16.15)	1,110(46.68)	1,898(79.81)	480 (20.19)
**Age**									
65-69		879(36.96)	721(82.03)	158(17.97)	379(43.12)	206(23.44)	294(33.45)	767(87.26)	112(12.74)
70-74		624(26.24)	463(74.20)	161(25.80)	246(39.42)	91(14.58)	287(45.99)	517(82.85)	107(17.15)
≥ 75		875(36.80)	467(53.37)	408(46.63)	259(29.60)	87(9.94)	529(60.46)	614(70.17)	261(29.83)
**Gender**									
Female		1,447(60.85)	900(62.20)	547(37.80)	547(37.80)	202(13.96)	698(48.24)	1,106(76.43)	341(23.57)
Male		931(39.15)	751(80.67)	180(19.33)	337(36.20)	182(19.55)	412(44.25)	792(85.07)	139(14.93)
**Education**									
Lower than primary school		1,585(66.65)	1,037(65.43)	548(34.57)	602(37.98)	282(14.32)	756(47.70)	1,233(77.79)	352(22.21)
Primary school or above		793(33.35)	614 (77.43)	179(22.57)	282(35.56)	157(19.80)	354(44.64)	665(83.86)	128(16.14)
**Area of residence**									
Rural		1,969(82.80)	1,370(69.58)	599(30.42)	712(36.16)	295(14.98)	962(48.86)	1,577(80.09)	392(19.91)
Urban		409(17.20)	281(68.70)	128(31.30)	172(42.05)	89(21.76)	148(36.19)	321(78.48)	88(21.52)
**Annual per capita household expenditure level**									
Q1^a^		625(26.28)	426(68.16)	199(31.84)	224(35.84)	82(13.12)	319(51.04)	458(73.28)	167(26.72)
Q2		650(27.33)	478(73.54)	172(26.46)	244(37.54)	99(15.23)	307(47.23)	524(80.62)	126(19.38)
Q3		510(21.45)	379(74.31)	131(25.69)	197(38.63)	81(15.88)	232(45.49)	431(84.51)	79(15.49)
Q4^b^		593(24.94)	368(62.06)	225(37.94)	219(36.93)	122(20.57)	252(42.50)	485(81.79)	108(18.21)

^a^Q1 the poorest, ^b^Q4 the richest

**Table 2:** Toileting assistance among older people with functional limitations. (Sample: older adults with functional limitations who also had a toileting disability; 226 persons)

**Factors**	**Toileting Assistance**		***p***
	**No**	**Yes**	
**Total**	121(53.54)	105(46.46)	
**Marital status**			
Couple	80(66.12)	72(68.57)	0.695
Single	41(33.88)	33(31.43)	
**Number of adult children**			
0 person	40(33.06)	49(46.67)	0.034
1-2 person	24(19.83)	10(9.52)	
≥ 3 persons	57(47.11)	46(43.81)	
**Live arrangement**			
With others	90(74.38)	95(90.48)	0.002
Alone	31(25.62)	10(9.52)	
**Age**			
65-69	40(33.06)	25(23.81)	0.114
70-74	34(28.10)	25(23.81)	
≥ 75	47(38.84)	55(52.38)	
**Gender**			
Female	78(64.46)	56(53.33)	0.089
Male	43(35.54)	49(46.67)	
**Education**			
Lower than primary school	77(63.64)	72(68.57)	0.435
Primary school or above	44(36.36)	33(31.43)	
**Area of residence**			
Rural	92(76.03)	83(79.05)	0.589
Urban	29(23.97)	22(20.95)	
**Annual per capita household expenditure level**			
Q1^a^	35(28.93)	22(20.95)	0.587
Q2	29(23.97)	27(25.71)	
Q3	29(23.97)	28(26.67)	
Q4^b^	28(23.14)	28(26.67)	

^a^Q1 the poorest, ^b^Q4 the richest

**Table 3:** Bathing assistance among older people with functional limitations. (Sample: older adults with functional limitations who also had a bathing disability; 377 persons)

**Factors**	**Bathing Assistance**		***p***
	**No**	**Yes**	
**Total**	48(12.73)	329(87.27)	
**Marital status**			
Couple	24(50.00)	224(68.09)	0.014
Single	24(50.00)	105(31.91)	
**Number of adult children**			
0 person	22(35.42)	135(41.03)	0.610
1-2 person	9(18.75)	46(13.98)	
≥ 3 persons	22(45.83)	148(44.98)	
**Live arrangement**			
With others	34(70.83)	277(84.19)	0.023
Alone	14(29.17)	52(15.81)	
**Age**			
65-69	21(43.75)	94(28.57)	0.034
70-74	14(29.17)	84(25.53)	
≥ 75	13(27.08)	151(45.90)	
**Gender**			
Female	28(58.33)	192(58.36)	0.997
Male	20(41.67)	137(41.64)	
**Education**			
Lower than primary school	36(75.00)	204(62.01)	0.080
Primary school or above	12(25.00)	125(37.99)	
**Area of residence**			
Rural	44(91.67)	256(77.81)	0.026
Urban	4(8.33)	73(22.19)	
**Annual per capita household expenditure level**			
Q1^a^	16(33.33)	88(26.75)	0.025
Q2	17(35.42)	76(23.10)	
Q3	11(22.92)	76(23.10)	
Q4^b^	4(8.33)	89(27.05)	

^a^Q1 the poorest, ^b^Q4 the richest

**Table 4:** Association between the availability of family care resources and toileting assistance in China. (Sample: older adults with functional limitations who also had a toileting disability; 226 persons)

**Factors**	**Adjusted Model**	***p***
	**OR 95%IC**	
**Marital status**^**a**^ (Reference: Couple)		
Single	1.56(0.50, 4.90)	0.439
**Number of adult children**^**b**^ (Reference: ≥ 3 persons)		
0 person	1.16(0.49, 2.76)	0.722
1-2 person(s)	0.28(0.09, 0.91)	0.034
**Live arrangement**^**c**^ (Reference: With others)		
Alone	0.39(0.10, 1.44)	0.161

^a^Model was adjusted for number of adult children, live arrangement, gender, age, education, area of residence, annual per capita household expenditure level, social activities, home and community-based services use, walking stick use, self-rated health status, number of chronic diseases, number of items in the difficulty in performing ADL category, number of items in the difficulty in performing IADL category

^b^Model was adjusted for marital status, live arrangement, gender, age, education, area of residence, annual per capita household expenditure level, social activities, home and community-based services use, walking stick use, self-rated health status, number of chronic diseases, number of items in the difficulty in performing ADL category, number of items in the difficulty in performing IADL category

^c^Model was adjusted for marital status, number of adult children, gender, age, education, area of residence, annual per capita household expenditure level, social activities, home and community-based services use, walking stick use, self-rated health status, number of chronic diseases, number of items in the difficulty in performing ADL category, number of items in the difficulty in performing IADL category

**Table 5:** Association between availability of family care resources and bathing assistance in China. (Sample: older adults with functional limitations who also had a bathing disability; 377 persons)

**Factors**	**Adjusted Model**	***p***
	**OR 95%IC**	
**Marital status**^**d**^ (Reference: Couple)		
Single	0.27(0.09, 0.78)	0.016
**Number of adult children**^**e**^ (Reference: ≥ 3 persons)		
0 person	1.33(0.43, 4.09)	0.618
1-2 person(s)	1.14(0.44, 2.93)	0.771
**Live arrangement**^**f**^ (Reference: With others)		
Alone	0.82 (0.32, 2.10)	0.694

^d^Model was adjusted for number of adult children, live arrangement, gender, age, education, area of residence, annual per capita household expenditure level, social activities, home and community-based services use, walking stick use, self-rated health status, number of chronic diseases, number of items in the difficulty in performing ADL category, number of items in the difficulty in performing IADL category

^e^Model was adjusted for marital status, live arrangement, gender, age, education, area of residence, annual per capita household expenditure level, social activities, home and community-based services use, walking stick use, self-rated health status, number of chronic diseases, number of items in the difficulty in performing ADL category, number of items in the difficulty in performing IADL category

^f^Model was adjusted for marital status, number of adult children, gender, age, education, area of residence, annual per capita household expenditure level, social activities, home and community-based services use, walking stick use, self-rated health status, number of chronic diseases, number of items in the difficulty in performing ADL category, number of items in the difficulty in performing IADL category

## Results

Overall, among individuals aged 65 and above with functional limitations, 69% had a spouse, 63% had at least one adult child, and 80% resided with family members. Spouse availability and living arrangement did not demonstrate statistically significant associations with toileting assistance. The availability of adult children and the living arrangement did not show statistically significant associations with bathing assistance. The following section provides detailed results.

### Availability of family care resources for older adults with functional limitations

#### Spouse availability

As shown in Table 1, the rate was lower for women compared to men (62.20% vs. 80.67%), for the 65-69 years old group compared to the ≥ 75 years old group (82.03% vs. 53.37%), for those living in rural areas compared to urban areas (69.58% vs. 68.70%), and for those with the lowest education level compared to the highest education level (65.43% vs. 77.43%).

#### Adult child availability

As shown in Table 1, approximately 46.68% had three or more adult children. Among rural older adults with functional limitations, around 63.2% had at least one adult child, and about 48.86% had three or more adult children. For urban older adults with functional limitations, approximately 57.95% had at least one adult child, and about 44.64% had three or more adult children.

#### Living arrangement

The rate was lower for women compared to men (76.43% vs. 85.07%), and was higher for those living in rural areas compared to urban areas (80.09% vs. 78.48%).

### Overview of toileting/bathing assistance received by adults with functional limitations who also have a toileting disability or bathing disability

About 46.46% of these adults with toileting disability received toileting assistance (Table 2). They were more likely to belong to the group with no adult children, live with other people, be female. Approximately 87.27% of these adults with bathing disability received bathing assistance (Table 3). They were more likely to belong to the group with a spouse, live with others, be in the ≥ 75 years old age group, have a lower level of education, reside in rural areas, and be in the highest annual per capita household expenditure level group.

### Association between availability of family care resources and toileting assistance or bathing assistance

The Variance Inflation Factors (VIFs) for the independent variables and covariates in models ranged from 1.07 to 2.76, indicating the absence of multicollinearity among the independent variables. Participants with 1-2 adult children had 72% lower odds of receiving toileting assistance, compared to those with ≥ 3 adult children (OR: 0.28, 95% CI: 0.09, 0.91, *p* = 0.034) (Table 4). However, spouse availability and living arrangement did not demonstrate statistically significant associations with toileting assistance. Participants who did not have a spouse had 73% lower odds of receiving bathing assistance (OR: 0.27, 95% CI: 0.09, 0.78, *p* = 0.016), compared to those who had a spouse (Table 5). However, the availability of adult children and the living arrangement did not show statistically significant associations with bathing assistance.

## Discussion

This study provides a national summary of availability of family care resources for older adults with functional limitations and assesses associations between availability of family care resources and bathing/toileting assistance among this population. The results showed that 69% of older adults with functional limitations had a spouse, and 63% of them had at least one adult child. Furthermore, 80% of them resided with others. These findings are consistent with previous research focused on older adults aged 60 years and above (77%) [20]. However, it is important to note that the availability of family care resources varied across demographic subgroups. For instance, the proportion of having a spouse in the female group (62%) was lower than that in the male group (81%). Similarly, the proportion of living with others in the female group (76%) was lower than that in the male group (85%). This phenomenon can be attributed to women’s longer life expectancy [34], which exposes them to the possibility of losing spouses and even their adult children, leading women to live alone. Furthermore, older adults in the 65-69 years old age group had a lower proportion of having ≥3 adult children available for support, compared to those in the ≥ 75 years old age group. This is also consistent with a gradual decline in fertility rates in recent decades in China.

Our study found that the prevalence of toileting disability was 14.17%, which was similar to the findings reported in an earlier study of the general population of adults aged 65 years and older (15%) [14]. Additionally, the prevalence of bathing and toileting disabilities among individuals aged 75 or older in China was 49% and 45%, respectively, which was higher than the prevalence of bathing and toileting disabilities among older adults aged 75 or older in the UK, which was reported as 34% and 17% [35]. One of the key findings in our study was that, overall, approximately 13% of older adults with bathing disabilities did not receive bathing assistance, and about 53% of these adults with toileting disabilities did not receive toileting assistance. These findings address the research gap concerning bathing and toileting assistance among older adults with functional limitations in China.

To our best knowledge, this is the first study to shed light on the association between availability of family care resources and bathing/toileting assistance among older adults in China. Our results revealed that participants who lacked a spouse had significantly lower odds of receiving bathing assistance, compared to those with a spouse. This highlights the importance of spousal relationships within the caregiving context and emphasizes the distinct role a spouse plays in providing bathing assistance as part of aging care. As we know, spouses often play a crucial role in offering emotional and physical support to their partners, which can be instrumental in maintaining personal hygiene and overall well-being [32]. However, adult child availability and living arrangement were not statistically associated with bathing assistance. One potential explanation for this phenomenon could be that the busy nature of livelihoods might hinder adult children from assisting their parents with bathing. Bathing is often considered one of the most demanding and disliked responsibilities for both caregivers and care recipients [28]; cohabitants may have a greater burden in facilitating bathing assistance. Another potential reason could be that family members do not perceive themselves as caregivers and fail to realize the need for proactive involvement in caregiving responsibilities. In other words, they not only lack the awareness of providing assistance with tasks such as bathing but also fail to recognize their role in participating in family care [36]. A study indicated that carers are often unaware of the supplementary responsibilities they have taken on until they confront a critical situation [36].

Our study also found that participants with 1-2 adult children had lower odds of receiving toileting assistance, compared to those with three or more adult children. However, spouse availability and living arrangement were not statistically associated with toileting assistance. The absence of this association could be attributed to the reason mentioned above—caregivers may not be able to identify their caregiving roles [37]. Another potential rationale is the potential reluctance of individuals with functional limitations to seek toileting assistance from constant cohabitants. Prior research indicates that toileting can evoke emotions of judgment, embarrassment, and shame [9]. Moreover, seeking aid for such personal tasks could detrimentally affect the self-esteem of disabled individuals [37, 38].

The implications of our findings hold policy significance. First, with the increasing prevalence of older adults with functional limitations, policymakers should thoroughly consider the requirements for toileting and bathing assistance among this demographic. Second, given that the majority of older adults with functional limitations reside with their families, we suggest that the government formulate comprehensive guidelines and allocate resources to prioritize supporting family care. As examples of commendable practices, local authorities can enhance the identification of carers and establish a comprehensive list of caregiving types. Moreover, engaging caregivers fully within a well-balanced framework that incorporates incentives, training, and respite service policies is imperative. These measures are necessary to enhance the efficacy of informal family caregivers and alleviate the burdens they face [7, 39]. Third, when older adults lack strong family support, it becomes crucial for affordable and accessible social care services to proactively reach out [7]. This is essential to fulfill this population’s requirements for bathing and toileting assistance.

### Limitations

Our study has several limitations. Firstly, our research design utilized a cross-sectional approach, enabling us to present a nationwide descriptive assessment of availability of family care resources, along with the association between availability of family care resources and receipt of toileting assistance or bathing assistance. However, it did not establish causality or offer a detailed account of how families decide to provide toileting assistance and bathing assistance over time, due to data availability and limitations associated with the CHARLS research design. To surmount these constraints and achieve deeper insights, we recommend adopting rigorously designed longitudinal studies in future endeavors. This longitudinal framework would facilitate a more profound exploration of caregiving dynamics within the context of evolving family support.

Secondly, it is notable that a for substantial proportion of those who received toileting and bathing assistance, their support cannot be singularly attributed to either a spouse or an adult child acting independently. This underscores the intricate involvement of multiple informal caregivers and diverse caregiver combinations, such as a spouse collaborating with an adult child, in furnishing aid to older adults throughout the progression of their condition. Comprehension of how family care is shared and harmonized among various caregivers becomes pivotal for the development of robust and comprehensive support systems tailored.

Thirdly, it is pertinent to acknowledge the absence of certain confounding variables in the CHARLS dataset, including factors relating to the intimate caregiver-care recipient relationship and the caregiver’s level of caregiving proficiency. The omission of these crucial elements may impact the interpretation of intergenerational caregiving arrangements and their sway on care outcomes. Hence, future research endeavors should consider integrating these crucial confounding variables to enhance analytical depth.

## Conclusion

In this study, the majority of participants had spouses, had at least one adult child, and lived with family members. Spouse availability and living arrangement did not demonstrate statistically significant associations with toileting assistance. The availability of adult children and the living arrangement did not show statistically significant associations with bathing assistance. Our findings suggested a gap in family commitment regarding aiding bathing/toileting for older adults with functional limitations. Governments should establish comprehensive guidelines and allocate resources to bolster family-centered care, including caregiver identification; list compilation; and engagement through incentives, training, and respite services. For those lacking robust family support, ensuring affordable and accessible formal care services becomes imperative to address bathing and toileting needs.

## Data Availability

The data used in this study were sourced from the China Health and Retirement Longitudinal Survey (CHARLS), a publicly accessible national database. The CHARLS survey data is openly available to the public for research purposes. The data that underpin the findings of this study, presented in aggregate form, are accessible from the primary author upon the submission of a reasonable request.
